# Long-acting reversible contraceptives (LARCs) as harm reduction: a qualitative study exploring views of women with histories of opioid misuse

**DOI:** 10.1186/s12954-021-00532-1

**Published:** 2021-08-04

**Authors:** Stephani L. Stancil, Melissa K. Miller, Alex Duello, Sarah Finocchario-Kessler, Kathy Goggin, Rachel P. Winograd, Emily A. Hurley

**Affiliations:** 1 Division of Adolescent Medicine, Children’s Mercy Kansas City, 2401 Gillham Rd, Kansas City, MO 64108 USA; 2 Division of Clinical Pharmacology, Toxicology and Therapeutic Innovation, Children’s Mercy Kansas City, Kansas City, MO USA; 3 Department of Pediatrics, University of Missouri-Kansas City, Kansas City, MO USA; 4 Missouri Institute of Mental Health, University of Missouri-St. Louis, St. Louis, MO USA; 5 Department of Family Medicine, University Kansas Medical School, Kansas City, KS USA; 6 Division of Health Services and Outcomes Research, Children’s Mercy Kansas City, Kansas City, MO USA; 7 School of Pharmacy, University of Missouri - Kansas City, Kansas City, MO USA

**Keywords:** Contraception, Long-acting reversible contraception (LARC), Opioid use disorder, Substance use, Unintended pregnancy

## Abstract

**Background:**

The sharp rise in opioid use disorder (OUD) among women coupled with disproportionally high rates of unintended pregnancy have led to a four-fold increase in the number of pregnant women with OUD in the United States over the past decade. Supporting intentional family planning can have multiple health benefits and reduce harms related to OUD but requires a comprehensive understanding of women’s perspectives of preventing unintended pregnancies. The purpose of this study was to comprehensively evaluate the knowledge, attitudes and experiences as they relate to seeking contraception, particularly LARCs, among women with active or recovered opioid misuse.

**Methods:**

In-depth interviews and focus group discussions with 36 women with current or past opioid misuse were recorded and transcribed. Transcripts were coded by ≥ 2 investigators. Themes related to contraceptive care seeking were identified and contextualized within the Health Belief Model.

**Results:**

Our analysis revealed seven interwoven themes that describe individual level factors associated with contraceptive care seeking in women with current or past opioid misuse: *relationship with drugs, reproductive experiences and self-perceptions, sexual partner dynamics, access, awareness of options, healthcare attitudes/experiences, and perceptions of contraception efficacy/ side effects.* Overall, perceived susceptibility and severity to unintended pregnancy varied, but most women perceived high benefits of contraception, particularly LARC. However, perceived barriers were too high for most to obtain desired contraception to support family planning intentions.

**Conclusions:**

The individual-level factors identified should inform the design of integrated services to promote patient-centered contraceptive counseling as a form of harm reduction. Interventions should reduce barriers to contraceptive access, particularly LARCs, and establish counseling strategies that use open, non-judgmental communication, acknowledge the continuum of reproductive needs, explore perceived susceptibility to pregnancy, and utilize peer educators.

## Background

In the Unites States, women with opioid use disorder (OUD) have rates of unintended pregnancy that are nearly two-fold higher than the general population [1–3] and the number of pregnant women with OUD has increased four-fold over the past decade [4]. Opioid misuse in pregnancy is associated with higher rates of adverse health outcomes such as pregnancy loss, and neonatal opioid withdrawal, a condition known as Neonatal Abstinence Syndrome (NAS) [5]. NAS increases risks of morbidity (e.g., low birthweight, structural abnormalities, neurological excitability, gastrointestinal dysfunction), medical service needs (i.e., intensive care admission and prolonged hospitalization) and long-term cognitive, learning and behavioral challenges [6, 7]. For mothers, unexpected fetal or neonatal death and NAS, (e.g., related child protection involvement and child removal) can contribute to significant emotional trauma and long-lasting mental health struggles leading to broader social and economic implications [8].

Most women with OUD report wanting to prevent pregnancy intention, but are less likely to use contraception than non-substance using peers at their last sexual encounter [2, 9]. As such, unintended pregnancy can be viewed as a harm associated with OUD, and promoting access to and use of reliable contraception as promising new direction for harm reduction among women who wish to avoid pregnancy. Many women with OUD report trying multiple contraceptive methods over time, yet, notably, use of highly effective long-acting reversible contraceptives (LARCs), like the implant and intrauterine device, is less common [1, 10–12]. Recent studies suggest that most women prefer LARCs over shorter-acting methods, but less than 1 in 5 have ever used one [1, 9–11, 13]. For those with low intention for LARC use, it remains unclear what specific perceptions may contribute [10].

Some studies have tried to provide clarity regarding reproductive health intentions and behaviors in women with substance use. Most individuals with substance use often delay or avoid seeking healthcare due to access barriers or fear of being shamed [14]. Regarding accessing reproductive healthcare specifically, many women with substance use describe numerous barriers such as lack of transportation, lack of knowledge of available health services, fear of law enforcement or child protective services involvement. Surveys focused on women with OUD suggest that limited contraceptive knowledge, fear of side effects or misconceptions about drug-use induced infertility may contribute to underutilization of contraception [1, 10, 11, 15]. Potentially influenced by sexual partner dynamics and trauma (e.g., intimate partner violence), pregnancy intentions in women with OUD may also be fluid and complex and impact desire for contraception [10, 16–22]. However, there remains a gap in knowledge of individual-level factors influencing women’s views of LARCs specifically in women engaged in active drug use.

As a part of broader opioid response efforts, the American Academy of Pediatrics and the American College of Obstetrics and Gynecology have endorsed expanding comprehensive contraception access, including LARC, as an essential harm reduction tool in the broader opioid epidemic response [23]. Although some pilot programs have begun to investigate strategies, large-scale expansion has yet to be fully realized [24, 25]. As programs are established, an in-depth understanding of individual-level factors related to sexual and reproductive health is necessary to support patient-centered care. This study aims to comprehensively evaluate the knowledge, attitudes and experiences regarding contraception, particularly regarding LARCs, among women with active or recovered opioid misuse. Findings from this study will generate a foundational understanding to inform patient-centered contraceptive provision for women with opioid misuse who wish to prevent pregnancy.

## Methods

### Setting and participants

We conducted this prospective, mixed-methods study in two large cities, one mid-size city, and surrounding rural communities in Missouri. Participants were recruited from service organizations within the state’s opioid response network (such as federally qualified health centers, substance abuse treatment programs, and syringe exchange programs). English-speaking women aged 18–45 years with self-reported current or recent misuse of opioids, subsequently referred to as opioid misuse, for which they were seeking substance use treatment or harm reduction (e.g., syringe exchange). The study was approved by the Institutional Review Board at Children’s Mercy Kansas City.

### Data collection

Data were collected in two phases: (1) in-depth interviews, intended to generate themes in an iterative process and (2) focus group discussions, for consensus and member-checking confirmation of themes [26]. For interviews, we employed a purposive sampling strategy aimed to obtain a demographically diverse group of women from urban/suburban/rural areas, with past/present opioid use, with high/low engagement with service organizations, and varied experience with pregnancy/childbearing. For focus groups, we recruited women ages 18–45 living in two different substance use recovery housing programs that accept women in early recovery, often with dual diagnoses (e.g., substance use and other mental health disorders) and provide ancillary support (e.g., faith-based meetings, support groups, education and employment assistance). There was no overlap in interview and focus group participants. All participants gave verbal informed consent and received a $25 gift card.

Interviews were approximately 60 min in duration. Interviewers used open-ended questions from a semi-structured guide based on by previously identified facilitators and barriers to contraception access and also explored emergent themes introduced by the participant [27]. Interview participants also answered closed-ended electronic survey questions regarding their demographics and substance use/reproductive health history. To establish trustworthiness through member-checking, focus group guides were designed to elicit feedback on preliminary findings from in depth interviews and inform remaining discussions [26].

### Data analysis

Analysis of interview transcripts was conducted concurrently with data collection. An iterative process informed subsequent data collection, as the team adjusted sampling priorities and interview content to address gaps [28]. Interviews and focus groups were audio-recorded, transcribed, censored of identifying information, and uploaded into Dedoose (Dedoose Version 7.0.23, Los Angeles, CA: SocioCultural Research Consultants, LLC). Transcripts were coded by ≥ 2 investigators. Themes were identified and examined alongside the Health Belief Model, [29] a theoretical framework that describes preventative health behaviors as intentional processes influenced by *perceived susceptibility* to developing the health problem, *perceived severity* of the consequences of the health problem, *perceived benefit* versus *perceived barriers* to engaging in the preventative behavior, *cues-to-action* that prompt the behavior and *self-efficacy,* or the individual’s perception of their own ability to perform the behavior.

## Results

### Interview participants

Fifteen women with current or past misuse of opioids were interviewed (Table 1). Over half (60%) had public health insurance and one-third (33.3%) had no insurance. None were actively seeking pregnancy and most (85.7%) were trying to avoid pregnancy. Over half (57.1%) did not use any method to prevent pregnancy during last vaginal sex. Of those who shared their pregnancy history, 77% (10/13) reported a past pregnancy. An additional 21 women in OUD recovery participated in the two focus groups conducted at residential recovery programs.

**Table 1:** Participant characteristics of those who completed in-depth interviews (n = 15)

Characteristic	Frequency (%)
Age category	
18–24	2 (13%)
24–34	7 (47%)
35–44	6 (40%)
Race	
White or Caucasian	13 (87%)
Black or African American	2 (13%)
Ethnicity	
Hispanic	4 (27%)
Educational attainment	
Some high school	5 (33%)
High school graduate or GED	2 (13%)
Some college or post high-school training	5 (33%)
Undergraduate degree	2 (13%)
Missing*	1 (7%)
Opioid use status	
In active use	5 (33%)
In recovery	10 (67%)
Residence	
Rural	2 (13%)
Suburban	7 (47%)
Urban	6 (40%)
Relationship status	
Married or domestic partnership	4 (27%)
In a committed relationship	1 (7%)
Single	10 (67%)
Current number of sexual partners	
None	8 (53%)
One	5 (33%)
More than one	1 (7%)
Missing/ declined	1 (7%)
Contraception use at last sex	
None	8 (53%)
Withdrawal	1 (7%)
Condom	3 (20%)
Birth control pill	1 (7%)
Injection	1 (7%)
Sterilization	1 (7%)
Condom at last sex	
Yes	2 (13%)
No	12 (80%)
Missing*	1 (7%)

^*^Not answered

### Overview of results

**Fig. 1:**
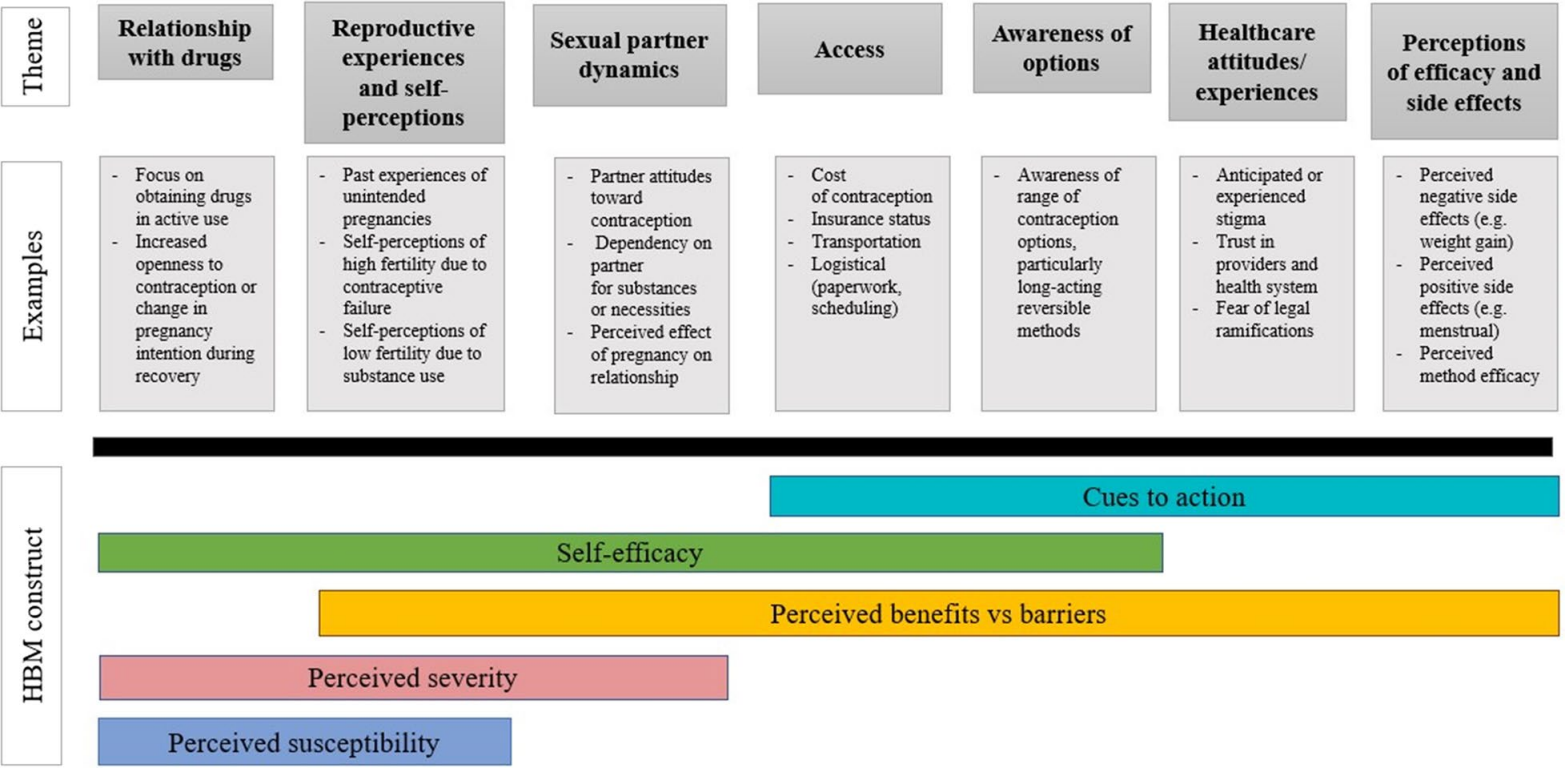
Relationship of overarching themes with Health Belief Model constructs

We identified seven overarching themes related to the views of contraception, specifically LARC, among women with opioid misuse: *relationship with drugs, reproductive experiences and self-perceptions, sexual partner dynamics, healthcare attitudes/experiences, access, awareness of options, and perceptions of contraception efficacy/side effects.* These themes were contextualized within the Health Belief Model (HBM) (Table 2, Fig. 1) to explore the connection with behavioral intention [11, 30–33].

**Table 2:** Examples of interconnectedness of themes and Health Belief Model constructs

Illustrative quotes	Themes	HBM constructs
“I don’t worry about getting pregnant because I haven’t been able to, according to doctors.”	Reproductive experiences and self-perceptions Healthcare attitudes/experiences	Perceived susceptibility to unintended pregnancy
“[Women with OUD] don’t want to have any more children because they’d get them taken away… I don’t want to have another one and think I can keep it, and one relapse means I never see it again.”	Relationship with drugs Reproductive experiences and self-perceptions	Perceived severity of unintended pregnancy
“I was just like, oh, I just wish I could get pregnant so he'll keep me forever. He won't hurt me no more. Maybe he'll love me more if I had his baby.”	Sexual partner dynamics	Perceived severity of unintended pregnancy
“So it makes it really hard when they have expectations of you and want you to have your insurance cards, and you have to have this or this, and you ain't got it because you've been pick-pocketed. But yet, it's like they're saying, ‘Well, we care about your health, but we don't care about your health because you ain't got insurance, so we can't help you.’”	Access Healthcare attitudes/experiences	Perceived barriers to contraception
“But then other girls who have been through it (pregnancy), they want birth control. But they're like me; they don't know anything about birth control. They don't know what kind of doctors. They don't have financial means to see a doctor. They don't even know how much it even costs to get birth control”	Awareness of contraception options Access	Perceived barriers to contraception
“It’s (the implant) the best thing that ever happened to me. It’d be perfect for a woman in recovery because if we relapse…I’m not going to go to the doctor.”	Reproductive experiences and self-perceptions Awareness of contraception options Relationship with drugs	Self-efficacy for contraception
"Everybody that I know in my family that have had their tubes tied, it only went right after the baby. Everytime. …And this piece of it (long-acting reversible contraception) had been an option, and I was trying to get clean, then maybe I would have thought about it."	Reproductive experiences and self-perceptions Access Awareness of contraception options	Cues to action for contraception care-seeking

Excerpts were chosen to illustrate the interconnectedness of the themes and HBM constructs. They are not meant to solely define each theme or construct

*HBM* Health Belief Model

### Relationship with drugs

Participants discussed how their *relationship with drugs* in active use and recovery affected their contraception perceptions and intentions. Active use, they described, is a “pretty demanding lifestyle” where “you do what you have to do to get the drugs”. With mental energy concentrated on “chasing the next high,” participants looking back on their time in active use claimed that they were not spending “enough time in reality” to think about sexual risks or going through the steps required to get birth control or health services. For many, active use reduced risk perceptions associated with unprotected sex (e.g., “It (pregnancy) never crossed my mind because I’m using. In my brain, I’m just thinking, ‘Oh, it’s just sex.’”). Some also described being “too full of pride” or too “paranoid” to seek healthcare. Participants generally considered LARCs valuable in active use, giving women “one less thing for me to worry about.”

In recovery, women began making looking at their health and future more intentionally. Some considered themselves better prepared for a pregnancy, including those whose pregnancy catalyzed their entry into recovery (“as soon as I found out I was pregnant, I was done”). Most in recovery, however, still believed an unintended pregnancy would bring challenges, preferring to wait until, “I have a stable living environment…so I don’t have to worry about feeding, and diapers” or “a non-abusive relationship and more support with trauma.” Some also acknowledged cyclic relationship with drugs, wanting to avoid pregnancy in recovery because, “you will probably relapse, more likely than not.”

### Reproductive experiences and self-perception*s*

Women reported varying *reproductive experiences and self-perceptions* that influenced diverse levels of perceived susceptibility to unintended pregnancy and the potential value of contraception. Many felt substance use had compromised their fertility (e.g., “using opiates does prevent you from getting pregnant”). Some who felt pregnancy was unlikely described amenorrhea during substance use, long periods of unprotected sex with no resultant pregnancy, or having been told by a health care provider that pregnancy was unlikely.

Perceptions of heightened susceptibility to pregnancy was rarer, but evident in women with a history of unintended pregnancies or a recent pregnancy scare. These experiences led some to consider contraception or sterilization, but others to doubt efficacy of contraceptive measures in general (e.g. “…even if you are being careful, it (pregnancy) can still happen”) or specifically for themselves (e.g. “birth control doesn’t work for me.”).

### Sexual partner dynamics

Women described how sexual partner dynamics could be a facilitator or a barrier to contraception care seeking. While some had partner(s) who were supportive of contraception, others experienced barriers from their partner’s desire for children. Some women perceived advantages to pregnancy for preserving or mollifying a relationship. Women often referred to dependent or abusive relationships in active use, where reliance on a partner for shelter, drugs, or food compromised women’s ability to seek measures to reduce sexual risks.

“I feel like when you're in addiction and you're with a man that feeds you drugs, you really don't care what you're going through, or the abuse…So, it really doesn't matter if you get pregnant or not.”

Further, women who earned money for sex often had to forfeit control over contraception to their “pimp” or partner, who could facilitate or restrict access.

### Healthcare attitudes/experiences

Women who had positive experiences with healthcare were more open to seeking reproductive health services. Women felt that identifying a provider whom they are “willing to trust,” comes off as “non-judgmental” and “there to help” was critical to ensure comfort in discussing their reproductive health and pregnancy intentions. They explained how the power of “word of mouth” recommendations from peers could identify trusted health service providers/locations and propagate perceived benefits, or conversely, serve as a barrier if negative experiences were shared. Women valued patient-centered communication with the primary focus being “to make sure you’re safe and you’re healthy.”

Many, however, recounted previous trauma with reproductive healthcare, making them reluctant to seek it. Some experienced pressure to take a certain type of contraception, for example, being told, “This is what everybody takes, so you’re going to” or, being refused a desired method, such as tubal ligation for being “too young.” Others recalled experiences with stigma in the healthcare setting, including one woman in active use who described feeling automatically judged because of her tattoos and “junkie” appearance.

Women believed engagement with peer liaisons could help facilitate more positive perceptions of contraception-related health care. Peers (e.g. “someone that has gone through it”) could help “make seeing a doctor less intimidating,” increase patient comfort, facilitate discussion of sensitive topics and improve trust.

### Access

Access barriers, particularly for LARC and prescription contraception, were numerous and often shaped by *healthcare attitudes/experiences*. In active use, participants were rarely connected to a primary care or women’s health provider, and many did not know where to go to obtain contraception services. For some, a syringe exchange program might be their only point of contact with healthcare. Those connected to healthcare were typically seeing specialists for substance use treatment and were rarely asked about sexual health or contraception. Women also described financial barriers amplified by economic instability, such as prohibitive cost of certain methods, lack of insurance coverage, lack of awareness of insurance or low-cost options, or barriers obtaining necessary prerequisites (e.g., homelessness letter, insurance/financial assistance application) required to access free/low-cost care. Perceived access barriers also included lack of transportation or ability to pay for transportation, and difficulty keeping healthcare appointments when actively using opioids.

Women in recovery cited difficulty in managing “a gazillion different doctors” along with school, work and parenting, favoring an “I can take care of that” approach from trusted provider they may be already seeing. Many did feel that recovery centers or syringe exchange programs could integrate on-site access to contraception, including LARC. "Oh, it's going to take two seconds and I'm going to get some birth control? Hell, yeah!" Another woman explained, “If I had had access like that, that was super simple and fast, I would have done it (LARC).”

### Awareness of options

Awareness of contraceptive options and how to obtain them varied for women. The majority of women in this study were more familiar with condoms, pill and injection and less familiar with LARC. After learning about LARC, women who were initially less aware liked that they would “be easier than remembering to take a pill every day” and “be better than having to go every three months to get a shot”. In the context of a demanding relationship with drugs, LARCs gave women in active use “one less thing for me to worry about.” For those early in recovery, “we're trying to get our head back, let alone not worrying about, did I take this pill? Did I not take this pill?”.

For some women who had previously been pregnant, they recalled getting “tubes tied” which was emphasized as “quick and easy” in the postnatal hospital setting. For many, this was the only option for preventing pregnancy that a healthcare provider had offered to them, and some regret that they did not receive education about other long-acting, yet reversible options. When discussing how to increase awareness of options, women preferred learning about options from a peer (someone with a history of addiction) or from a health care provider recommended by a peer.

### Perceptions of contraception efficacy/side effects

Women had varied perceptions of contraceptive efficacy and side effects. Some women expressed fears of compromising future fertility or potentially harming the fetus if pregnancy occurs while using contraception. Misconceptions about drug-drug interactions were also common (e.g., meth or cocaine causing more harm in combination with hormonal birth control). Women generally perceived valuable benefits of LARCs including the allowance of reversible “long-term” and “short-term” family planning. Yet some feared perceived invasive or complicated procedures required to obtain LARCs (e.g. “I don’t want anything in me”) or worries the implant may be a “tracking device.” One woman got the implant removed after a year because it “freaked (me) out” and subsequently got pregnant while taking oral contraceptive pills. Others were deterred by personal or anecdotal experiences, specifically related to LARCs (e.g., menstrual changes, device breakage, intrauterine device (IUD) movement/expulsion, IUD related infections, partner feeling IUD during sex, perceived ineffectiveness). Many also described positive and negative side effects (e.g., weight gain, amenorrhea, reduced menstrual symptoms and reduced acne) of a specific hormonal contraceptive method, like pills, and projected those same ideas to contraception overall, including LARC. Most women did not express understanding of the benefits and risks of individual methods and how they compare to one another. Most women felt that educating about individual contraceptive methods and dispelling myths regarding efficacy and side effects would best be accomplished through the use of a peer educator.

## Discussion

Contraception use among women who wish to prevent pregnancy is a potential means of reducing harms associated with opioid misuse, namely the increased risk of experiencing an unintended pregnancy and of complications for both the fetus and mother. Therefore, improving access to patient-centered contraception provision for women who desire it, regardless of their choices regarding opioid use, represents a promising harm reduction strategy. Our study provides essential context for harm reduction efforts aiming to leverage this strategy by illuminating individual- level factors at the intersection of contraceptive care-seeking and opioid use. Among our sample of Midwestern women with current or recent OUD, contraceptive care seeking was influenced by *relationship with drugs, reproductive experiences and self-perceptions, sexual partner dynamics, access, awareness of options, healthcare attitudes/experiences, perceptions of contraception efficacy and side effects.* These interconnected themes aligned with constructs of the Health Belief Model (HBM), which has been widely applied to explain contraception intentions and inform interventions to promote reproductive autonomy [11, 30–33].Contextualized within the common HBM “language,” the themes identified in our study provide deeper meaning, layer by layer, specific to women with opioid use. Our findings augment the existing literature to reveal that, despite the presence of contraception desire and even method preference in women with current or past opioid misuse, actual use is often impeded by high perceived barriers, poor self-efficacy, and limited cues to action, particularly for LARCs. Women in our study also share ideas for patient-centered, comprehensive reproductive health care that reduces harms of opioid misuse which, in turn, can facilitate improved quality of life.

Among the themes we identified, *access*, stands out as one of the most prominent barriers, suggesting system-level solutions are needed. When probing specifically about LARCs, women generally emphasized benefits, but believed access to be the biggest challenge. To address access, women recommended integrating reproductive health into their existing OUD care. Other themes, like *health care attitudes, reproductive experiences, awareness* and a dynamic *relationship with drugs* suggest benefits of providing comprehensive reproductive care as part of a harm-reduction strategy in settings like recovery centers and syringe exchange programs. Previous literature suggests women with OUD are open to reproductive health care co-occurring with substance use treatment [9, 15, 25, 34, 35]. Opioid-specific public health response has endorsed that comprehensive contraceptive provision, including LARC, is within the scope of practice for a wide variety of clinicians, yet education is needed to improve provider comfort and boost patient access [23].

Concurrent with system-level reform, contraception requires autonomous, intentional care-seeking. Contraceptive counseling and service design should be non-directive, patient-centered and informed by the themes identified in our study with the following strategies:

1. Utilize open, non-judgmental communication: Women’s descriptions of past *experiences with health care* and desire for future reproductive healthcare overwhelmingly emphasize this need and is consistent with a high perceived emotional cost of seeking care [36]. Given the high rates of trauma among women with OUD, trauma-informed care iis a useful counseling framework, particularly around issues related to sexual abuse, coercion, pregnancy loss or termination, and child custody loss that were present in our data and should be inherent in conversations related to reproductive health. Indeed, reducing emotional cost of seeking.
2. Acknowledge the continuum of reproductive needs in women with OUD, from pregnancy intention to contraception care-seeking. Our findings reveal multifaceted influences on individual reproductive needs including varying *sexual partner dynamics, reproductive experiences and self-perceptions* and *relationship with drugs*. The fear that desire for pregnancy in the setting of co-morbidities like substance use disorder may be or has been pathologized by a provider was expressed in our study and is seen in the literature[37]. Such negative health care experiences may prevent women from seeking reproductive care of any kind, reducing opportunity for health promotion, risk reduction counseling or contraceptive education.
3. Explore individual perceived susceptibility to unintended pregnancy: Women who are substance users are two times more likely to get pregnant if they hold views that they are “unhealthy” or perceive that they cannot get pregnant compared with those without these views [20]. In our study, some women had perceptions of reduced susceptibility to pregnancy similar to previous studies, including beliefs that amenorrhea signified lack of ability to get pregnant and thus negated any need for contraception [38]. On the other hand, some participants in our study believed they were overly susceptible, to the point that contraception would not work for them. Identifying individual perceived susceptibility with open-ended questioning can help create a more targeted, patient-centered counseling experience that better educates individual women about their choices.
4. Use a peer educator when possible to deliver patient education, particularly surrounding inaccurate information, fear of complications and distrust with healthcare. Women in our study and previous literature [11] described misconceptions regarding contraceptive risks or propagated third-party stories that inflated risks. Women in our study provide a solution for improved care: integrating “word of mouth” from a trusted peer to facilitate contraception seeking and use.

### Limitations

Our purposive sampling strategy was designed to promote inclusion of a diverse group of women; however, certain racial and ethnic groups were less represented in the demographics of the organizations and thus less represented in our data Although we do not understand all factors related to underrepresentation of minorities in our partner treatment centers, contributors may include differential experiences with stigma encountered when seeking substance use services [39–42] and preference for faith-based treatment in certain racial andethnic groups over medication assisted treatment [43, 44]. We were able to recruit some women in active drug use. Yet, future work employing targeted recruitment strategies for women in active drug use, specifically those who do not engage with any healthcare system, including syringe exchanges, wwould be important to provide a wide array of perspectives.

## Conclusions

A complex interplay of individual-level factors impacts contraceptive care seeking in women with current or past opioid misuse. Our findings suggest that reduction of barriers through efficient access and comprehensive patient-centered education may, in turn, improve reproductive health care experiences and support individual family planning goals. Suggestions from the women in our study describe their preference to empower peers to share contraceptive information and support one another with strategies that meet their needs. Interventions that address the core themes conveyed by women with opioid misuse may help facilitate patient-centered reproductive health care that meets women “where they are at” to reduce harms associated with opioid exposed unintended pregnancy.

## Data Availability

The de-identified datasets analyzed during the current study are available from the corresponding author on reasonable request.
